# Supplementary figures and images for: Coordinate genomic association of transcription factors controlled by an imported quorum sensing peptide in Cryptococcus neoformans
Source: PLoS Genet. 2020 Sep 21;16(9):e1008744. doi: 10.1371/journal.pgen.1008744 (PMC7537855; doi:10.1371/journal.pgen.1008744)

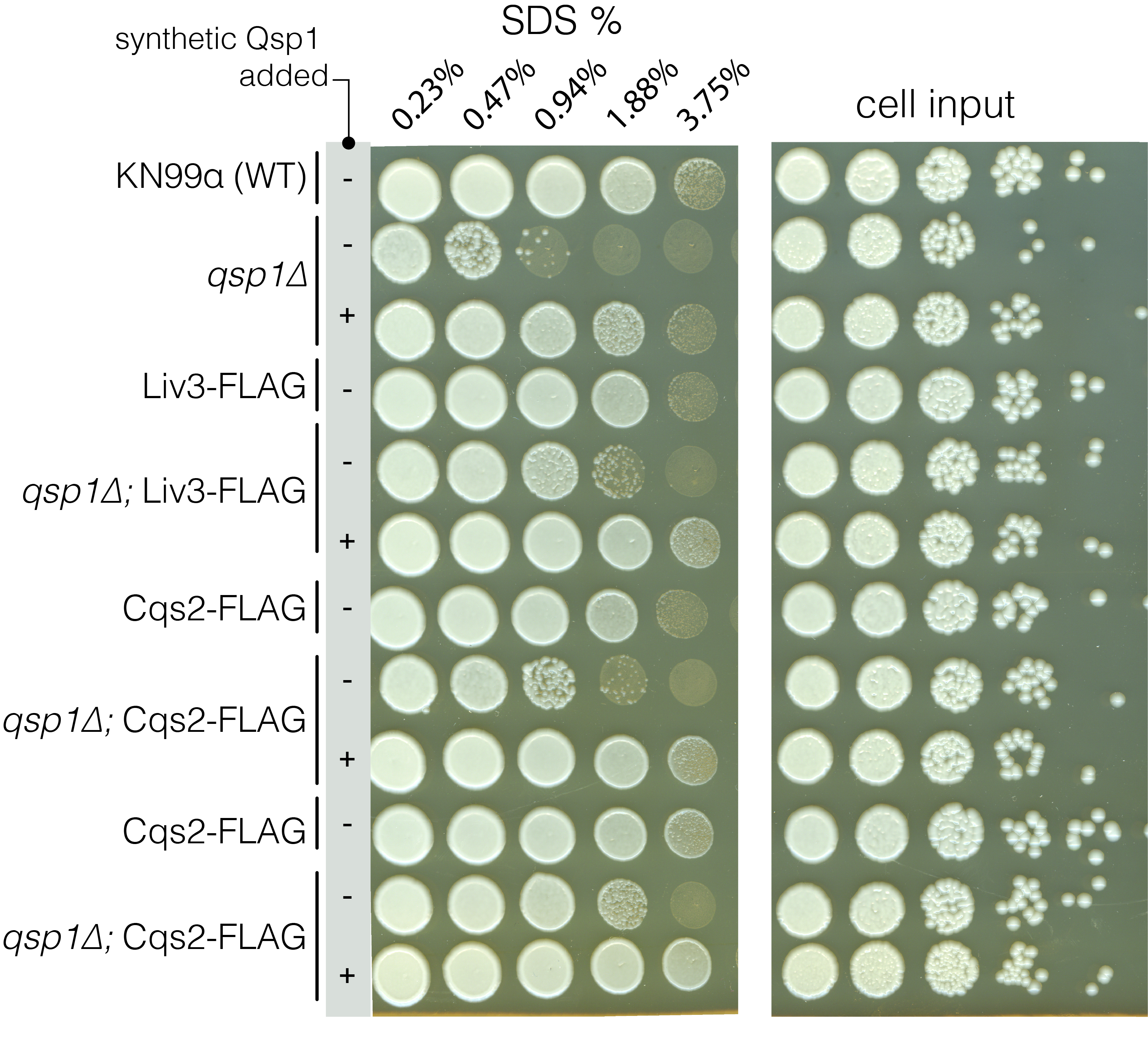

Supplement: S1 Fig — Each genotype shown was tested for their ability to survive increasing concentrations of the cell wall stressor SDS. 1 uM synthetic Qsp1 peptide was added to the indicated cultures (+) from the time of inoculation, or not (-). Water dilutions of each culture are shown to the right as a measure of cell input. Plates were allowed to grow up at room temperature for 4 days. (TIF) [file pgen.1008744.s001.tif]

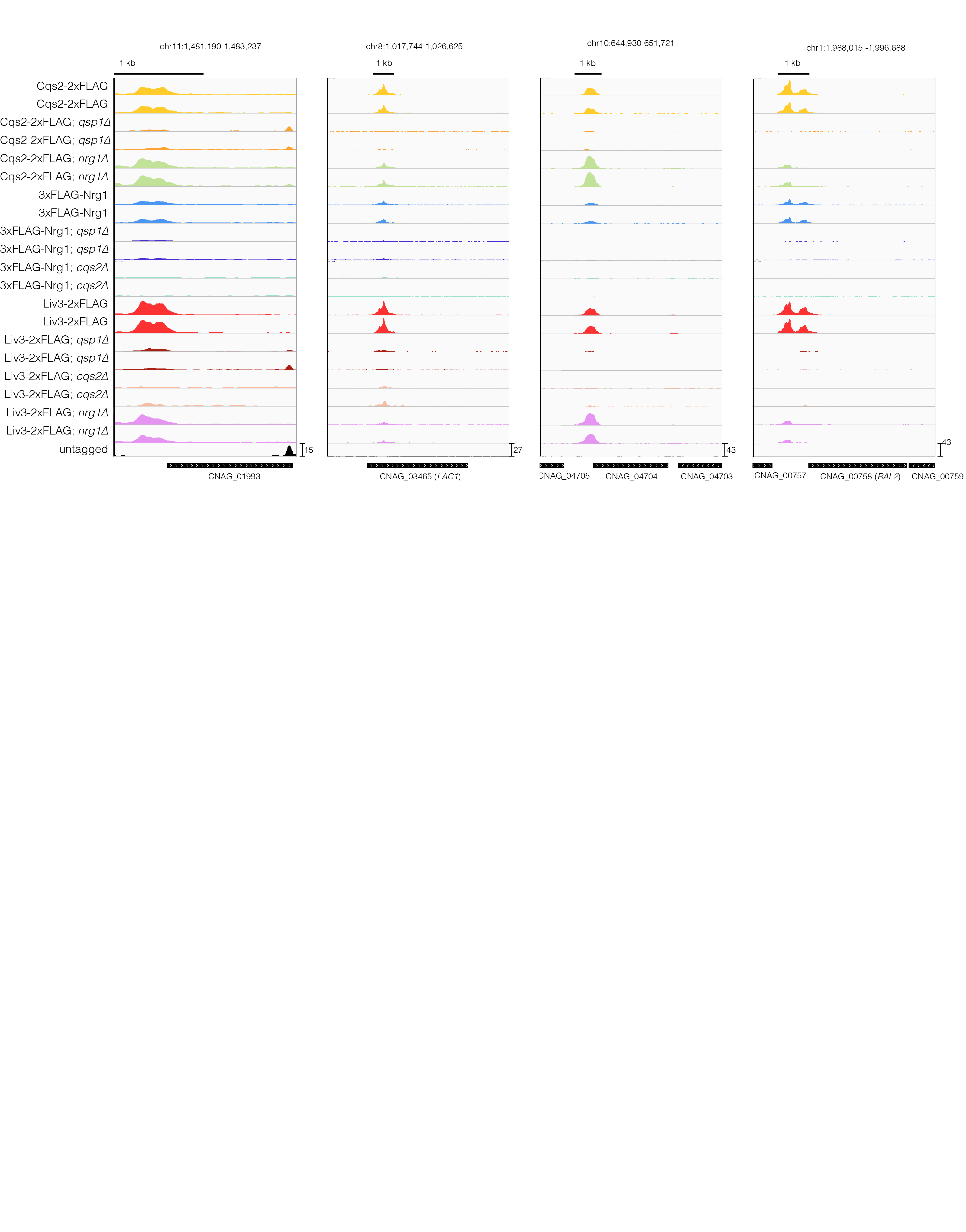

Supplement: S2 Fig — ChIP-seq data visualized using the Integrative Genomics Viewer software. Scale bars measuring 1kb are shown at the top of each screenshot, along with chromosomal location. Gene transcripts are shown in black boxes with white arrows showing directionality. (TIF) [file pgen.1008744.s002.tif]

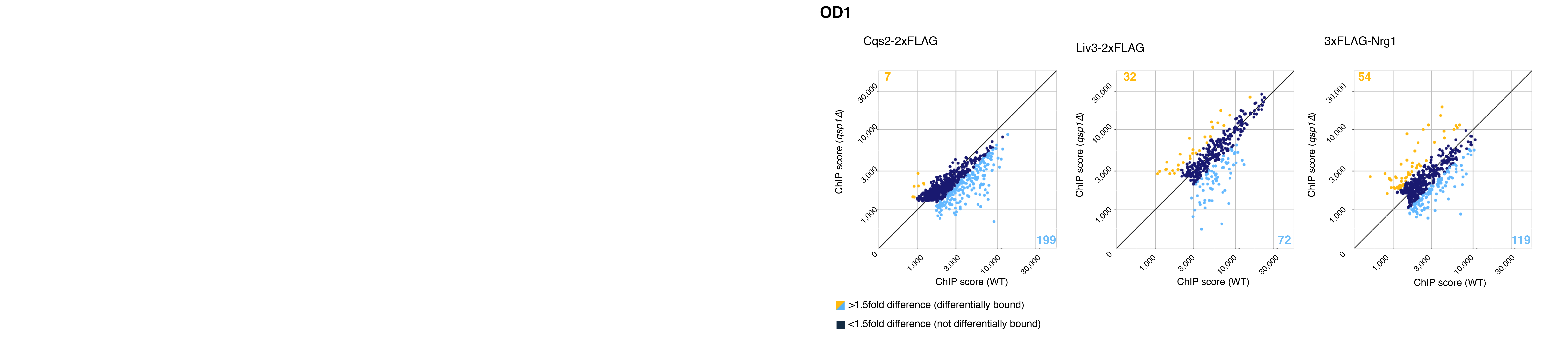

Supplement: S3 Fig — ChIP score for each gene was calculated as the read depth in the 1 kb region upstream of the transcription start site, normalized to the untagged control. Only promoters that are called as bound in either genotype by k-means analysis are shown, with promoters that are more or less bound (>1.5-fold changed) by each factor in the qsp1Δ mutant highlighted in orange or light blue, respectively. The number of promoters in either of these groups is labeled with the corresponding color. (TIF) [file pgen.1008744.s003.tif]

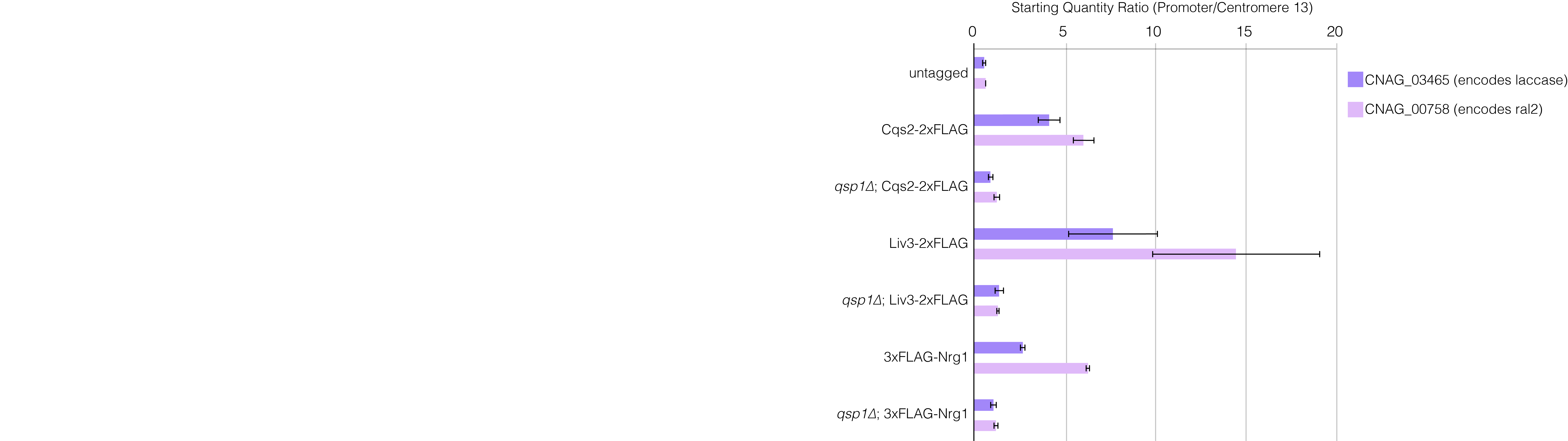

Supplement: S4 Fig — ChIP was performed on tagged strains followed by qPCR to quantify binding of CNAG_00758 and CNAG_03465 by tagged Nrg1, Liv3, and Cqs2 in wild type or qsp1Δ knockout using the primers in Table 4. (TIF) [file pgen.1008744.s004.tif]

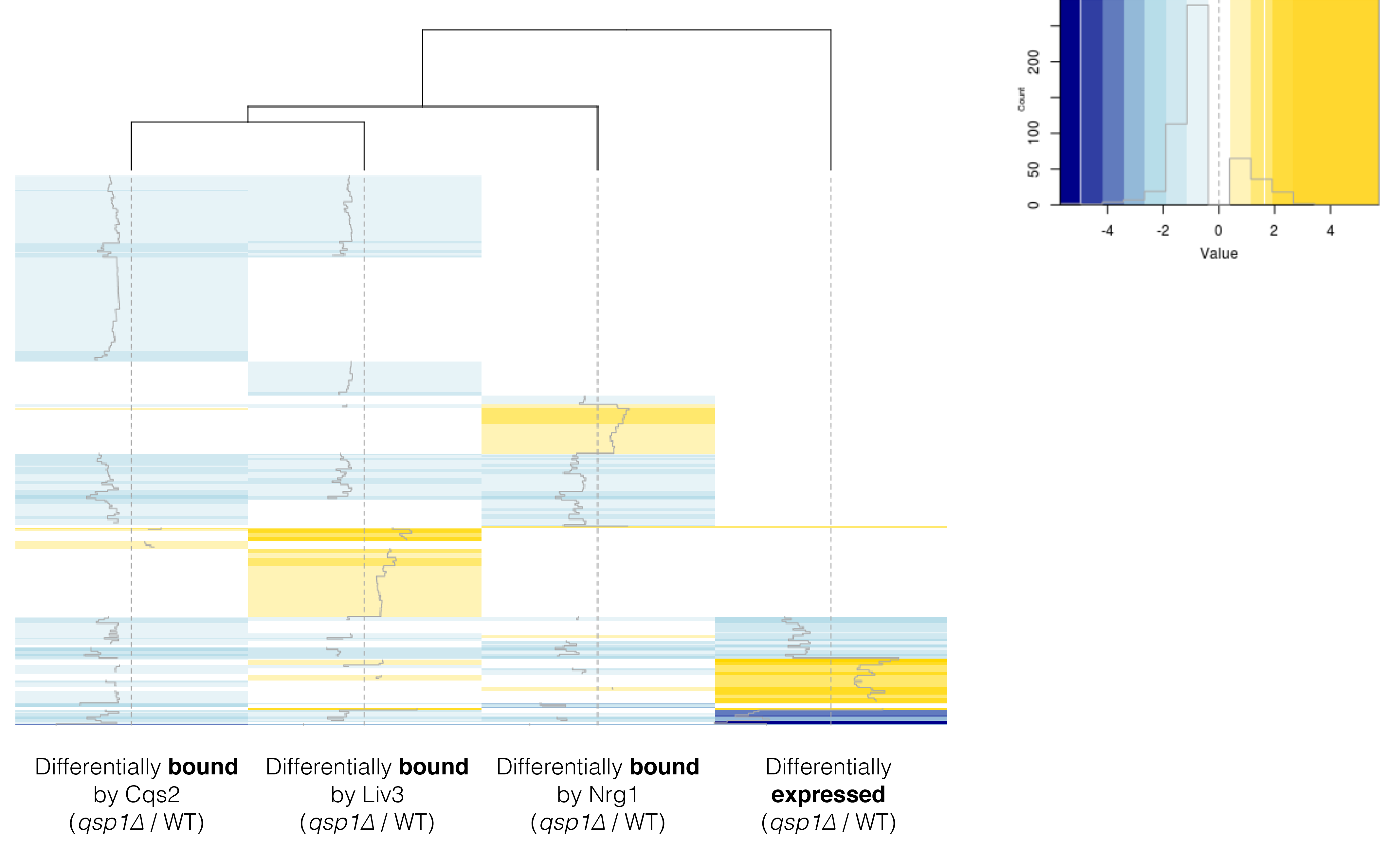

Supplement: S5 Fig — Non-significant differences are colored in white, significant decreases in mutant are shown in blue, and significant increases in qsp1Δ over wild type are shown in yellow. (TIF) [file pgen.1008744.s005.tif]

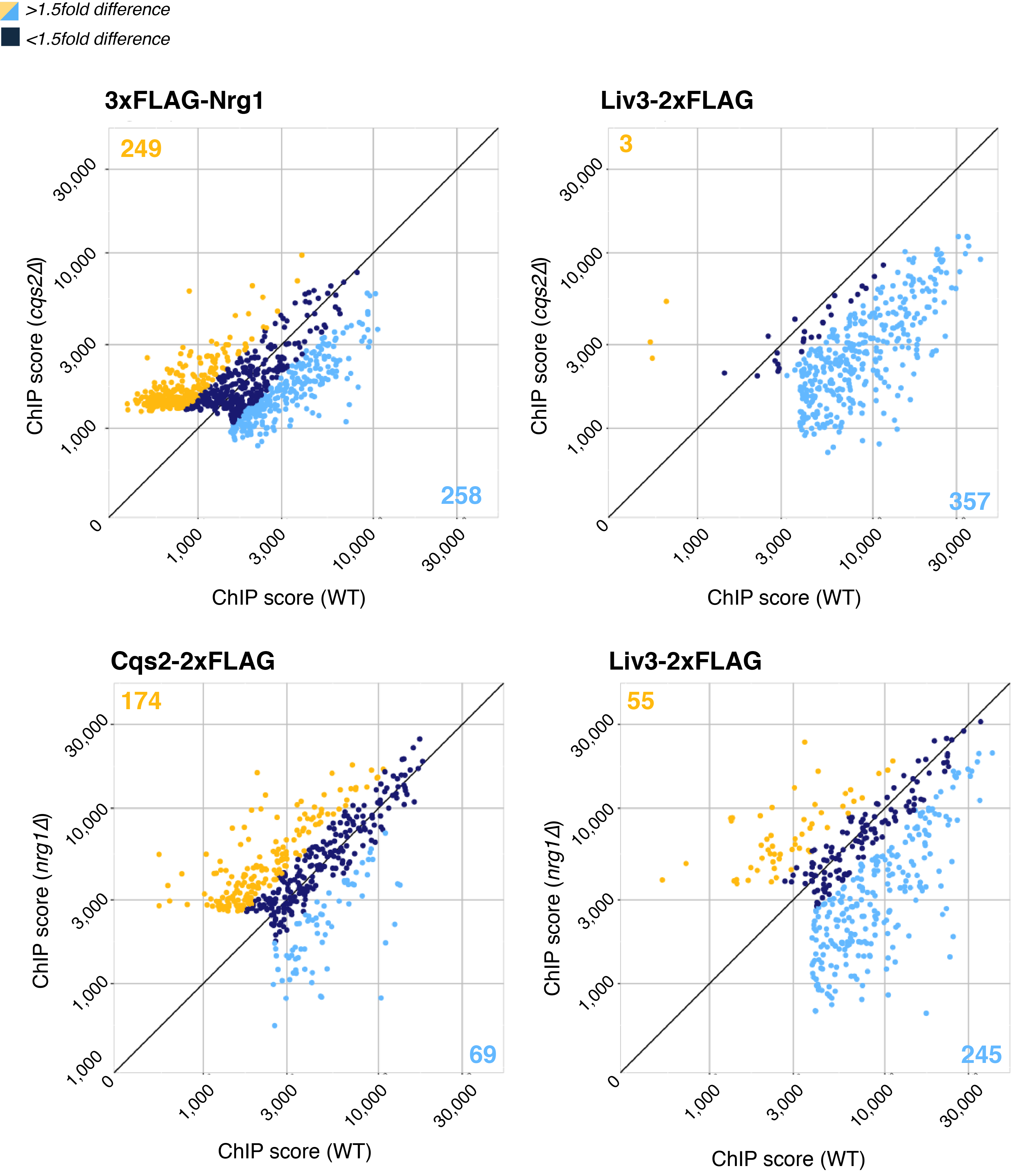

Supplement: S6 Fig — The ChIP score for each gene was calculated as the read depth in the 1 kb region upstream of the transcription start site, normalized to the untagged control. Only genes that are called as bound in either genotype by k-means analysis are shown, with genes that are differentially bound by each factor in mutant compared to wild type (greater than 1.5-fold changed) highlighted in light blue. (TIF) [file pgen.1008744.s006.tif]

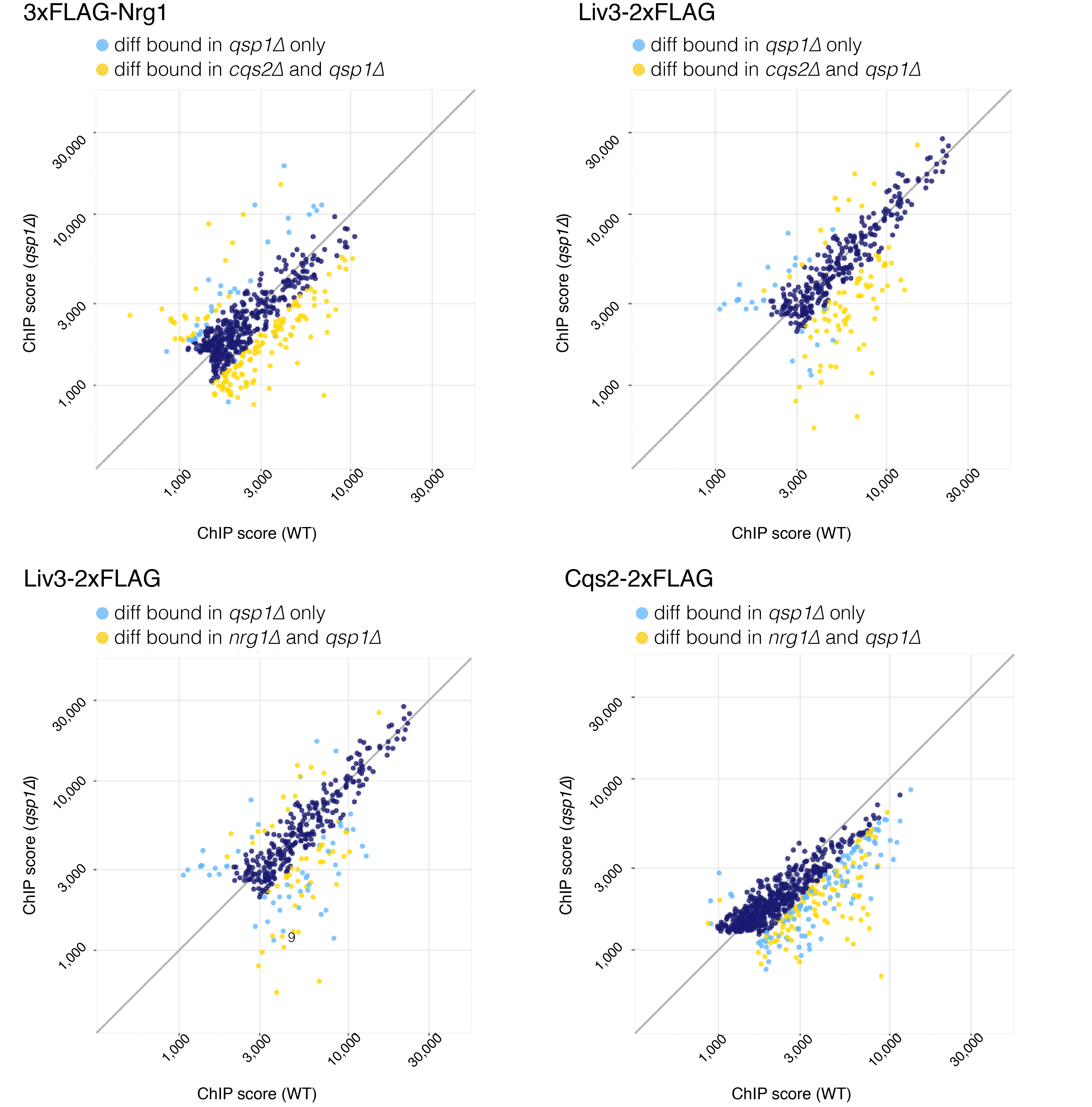

Supplement: S7 Fig — The ChIP score for each gene was calculated as the read depth in the 1 kb region upstream of the transcription start site, normalized to the untagged control. Only genes that are called as bound in either genotype by k-means analysis are shown, with genes that are differentially bound (>1.5-fold changed) by each transcription factor in the qsp1Δ mutant highlighted (light blue and yellow). Promoters that are differentially bound by each transcription factor in the transcription factor mutant are highlighted in yellow. (TIF) [file pgen.1008744.s007.tif]
